# Contour Erasure and Filling-in: Old Simulations Account for Most New Observations

**DOI:** 10.1068/i0684

**Published:** 2015-04-01

**Authors:** Gregory Francis

**Affiliations:** Department of Psychological Sciences, Purdue University, West Lafayette, Indiana, USA and Brain Mind Institute, École Polytechnique Fédérale de Lausanne, Lausanne, Switzerland

**Keywords:** contour adaptation, filling-in, brightness perception, boundary completion

## Abstract

Three recent studies used similar stimulus sequences to investigate mechanisms for brightness perception. Anstis and Greenlee (2014) demonstrated that adaptation to a flickering black and white outline erased the visibility of a subsequent target shape defined by a luminance increment or decrement. Robinson and de Sa (2012, 2013) used a flickering disk or annulus to show a similar effect. Here, a neural network model of visual perception (Francis & Kim, 2012), that previously explained properties of scene fading, is shown to also explain most of the erasure effects reported by Anstis and Greenlee and by Robinson and de Sa. The model proposes that in normal viewing conditions a brightness filling-in process is constrained by oriented boundaries, which thereby define separate regions of a visual scene. Contour adaptation can weaken the boundaries and thereby allow brightness signals to merge together, which renders target stimuli indistinguishable from the background. New simulations with the stimuli used by Anstis and Greenlee and Robinson and de Sa produce model output very similar to the perceptual experience of human observers. Finally, the model predicts that adaptation to illusory contours will not produce contour erasure.

## 1 Introduction

Anstis and Greenlee (2014) demonstrated contour adaptation effects on the perceived brightness of a subsequent target that was defined by small luminance increments or decrements from a gray background. The perceived contrast of the target stimulus was much reduced when a flickering outline contour was presented prior to the target, which they called an “erasure effect.” With a variety of demonstrations, they examined properties of adaptation, brightness filling-in, and the relation between these mechanisms and visual pop-out. For example, they noted that the erasure effect was dependent on the size of the adapting contour and the size of the test stimulus. Erasure occurred for equal-sized stimuli, but if the test stimulus was larger or smaller than the adapting stimulus there was little or no erasure. Anstis and Greenlee argued that their findings support the general idea of brightness filling-in being constrained by boundary contours. As described below, these ideas are part of an existing neural network model (Grossberg & Todorovic, 1988; Francis & Kim, 2012), and new simulations of the model show how they can be quantitatively instantiated.

The findings of Anstis and Greenlee (2014) are similar to earlier studies on flicker adaptation. Robinson and de Sa (2012) adapted observers to flickering disks of varying sizes and measured contrast thresholds for a flickering test disk. Thresholds were substantially increased only when the test and adapting disk had the same size. Similar results were found for a polar checkerboard adaptor, with thresholds increasing when the test disk contours were aligned with interior contours of the checkerboard. In further studies of this type, Robinson and de Sa (2013) adapted observers to a large flickering annulus, which induced perceived flicker of the annulus interior even though it was physically an unchanging gray. Observers then judged the polarity of a subsequent test shape located at the center of the annulus. Similar to Anstis and Greenlee and their earlier study, Robinson and de Sa (2013) found that a test stimulus the same size as the annulus interior was perceived to be of a lower contrast than an unadapted comparison stimulus. Reducing the size of the test stimulus to be smaller than the annulus interior eliminated the adaptation effect. Although the stimuli, experiments, and results are similar to those in Anstis and Greenlee (2014), Robinson and de Sa (2013) argued that these effects were inconsistent with a filling-in model of perception because adaptation caused by induced flicker should have had equivalent effects regardless of the test stimulus' size. As described below, new simulations of the Francis and Kim (2012) model provide an interpretation quite different from Robinson and de Sa (2012, 2013) and suggest that their findings strongly support the filling-in properties of the model.

Stephen Grossberg has long championed the idea that visual perception involves a filling-in process for brightness and color perception. An integral part of his theory is that boundary contours constrain the filling-in process with complementary pathways that trade-off limitations of different types of visual processing (Grossberg & Mingolla, 1985; Grossberg & Todorovic, 1988; Grossberg & Hong, 2006). In the model, anything that affects the strength of boundary contours, such as contour adaptation, also affects the filling-in process. Contour adaptation plays an important role in Grossberg's model. In particular, Francis, Grossberg, and Mingolla (1994) showed that contour adaptation helped control the duration of visual persistence that would otherwise be unacceptably long due to excitatory feedback in neural circuits. The same contour adaptation mechanism was shown to modulate the filling-in of visual afterimages (Francis & Rothmayer, 2003; Francis & Ericson, 2004; Francis & Schoonveld, 2005; Wede & Francis, 2006, 2007; Van Horn & Francis, 2007) and filling-in effects described as scene fading (Simons, Lleras, Martinez-Conde, Slichter, Caddigan & Nevearez, 2006; Francis & Kim, 2012). The model suggests that many of these investigations are related to the erasure effects noted by Anstis and Greenlee (2014) and Robinson and de Sa (2012, 2013).

For example, Experiment 1 in Francis and Schoonveld (2005) was designed to test a model prediction that a flickering oriented grating could modify the percept of an afterimage produced by a preceding grid pattern that contained both vertical and horizontal bars on a gray background. As predicted by the model, offset of a flickering vertical grating produced an afterimage percept of horizontal bars. Thus, the vertical bars that would normally be present in the retinal afterimage were rendered invisible by adaptation to the flickering grating. This influence is very similar to the contour erasure effect. Moreover, Francis and Schoonveld (2005) showed that contour adaptation leads not only to erasure (disappearance of the vertical parts of the afterimage) but also to construction of a new perceptual experience (visibility of complete horizontal bars from the incomplete parts of the afterimage). The observations of Anstis and Greenlee (2014) seem similar to the effects identified by Francis and Schoonveld, but differ in that the weak afterimage is replaced with a weak physical stimulus.

Similarly, Francis and Kim (2012) successfully simulated effects of scene fading (Simons *et al.*, 2006). In one version of scene fading, dots are randomly flashed over a blurry photograph. Over time, different regions of the photograph appear to merge together into a faded percept. Similar effects were produced by the offset (but not the onset) of static dots and by an abrupt drop in the photograph's contrast. Francis and Kim (2012) showed that a model based on contour adaptation and filling-in could account for almost all of the reported properties of scene fading. The explanation is nearly the same as for Francis and Schoonveld (2005): the adaptation stimulus weakens boundary contours and thereby allows brightness/color signals to spread via a filling-in process.

This article reports new simulations to show that the model simulation of Francis and Kim (2012) also accounts for most of the observations reported by Anstis and Greenlee (2014) and Robinson and de Sa (2012, 2013). The simulations reported here used the very same model (structure and parameters) as Francis and Kim (2012). Only the stimulus conditions changed for the new simulations. MatLab code to reproduce the simulations described in this article can be found at the Open Science Framework (https://osf.io/dve5w/?view_only=0bf4c5fc39884b1c9bb506e912c3aa7d).

## 2 Model description

This section describes the basic properties of the model that are related to contour erasure. Many characteristics of the model are also involved in other aspects of visual perception, so what is described here is only one perspective of the model, its properties, and the reasons for those properties. The model proposes that the visual system contains two complementary and interacting pathways (Grossberg & Mingolla, 1985). A Boundary Contour System processes oriented contour information that represents edges of stimuli and perceptual groups between elements in a scene (such as illusory contours or texture patterns). The information in the Boundary Contour System also constrains a filling-in process in a Feature Contour System that represents brightness and color information across a surface.

**Figure 1.**
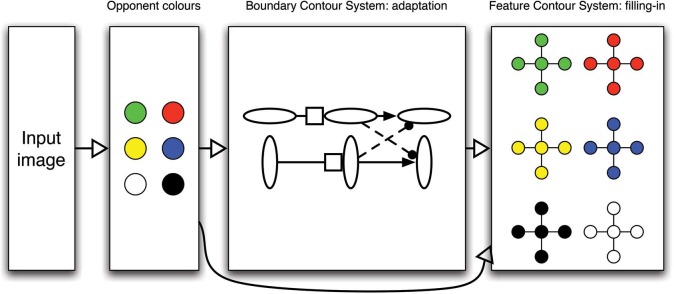
Each box represents processing at a pixel by schematizing the circuits related to contour adaptation and filling-in. Contour adaptation occurs in the Boundary Contour System as part of a gated dipole circuit for cells sensitive to orthogonally oriented boundaries. Oriented boundaries are necessary to constrain brightness/color signals in the Feature Contour System that otherwise spread freely to neighboring pixels. Such constraints can disappear due to contour adaptation, which renders parts of the scene invisible.

Figure 1 schematizes the main components of the model that are related to contour adaptation and filling-in. The input image is partitioned into parallel pathways that code complementary colors. These pathways then feed into circuits that process oriented boundaries. Figure 1 schematizes neurons that code horizontal and vertical orientations at a given pixel. These orientation-sensitive neurons form a circuit called a gated dipole (Grossberg, 1972), which is the source of contour adaptation in the model.

In the gated dipole circuit schematized in Figure 1, information flows from left to right. In addition to input from the opponent color cells, each orientation-sensitive neuron on the left side of the circuit also receives a constant common input, which drives after-responses at higher levels of the circuit. Activity from the first level to the second level passes through habituating neurotransmitter gates (indicated by the squares). Signals passing from the first level use a proportional amount of neurotransmitter to communicate to the second level; and over time the gate depletes and thereby becomes less effective at passing along the information. Typically, this depletion process is modeled by a multiplication between the activity of neurons at the first level and the gate strength. The gate strength changes as a mass action differential equation (details are in Grossberg, 1972; Francis et al., 1994; and Francis & Kim, 2012). Connections from the second to third layer of the gated dipole circuit include excitation within the same orientation and inhibition (dashed lines) across orthogonal orientations. Thresholded activity of this third layer acts as the “output” of the gated dipole circuit at a given pixel. The gated dipole circuit is repeated at each pixel in the image plane.

**Figure 2.**
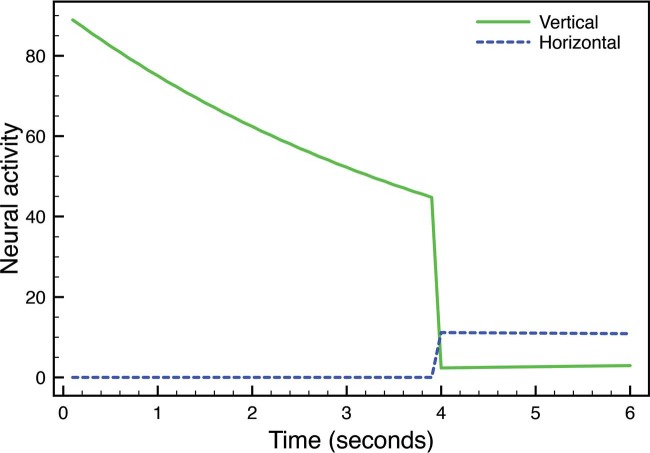
Activity of oriented boundary signals within a gated dipole circuit as a function of time. A vertical edge was presented for 4 s, and the vertically tuned boundary responds strongly and then fades due to adaptation. At time 4, the strong vertical edge is replaced by a weak vertical edge, and the drop in input leads to a rebound of activity in the nonstimulated (horizontally tuned) boundary.

Figure 2 plots the unthresholded activity of the vertical and horizontal cells within a gated dipole circuit at a pixel location that responded to a strong vertical edge for 4 s and then a much weaker vertical edge for 2 s. From time 0 to 4, Figure 2 shows that the activity for the vertical cell decreases, which occurs because of depletion of the vertical pathway's transmitter gate. At time 4, the vertical cell activity drops dramatically due to the strong edge being replaced by a weak edge. Since a depleted transmitter gate weights the signal for the vertical edge, it sends below normal inhibition to the horizontal cell. This reduced inhibition allows the common tonic input to the horizontal pathway to generate an after-response. As the vertical pathway's transmitter gate gradually recovers in response to the lower input level, the cross-orientation inhibition will become stronger and the horizontal after-response will disappear.

**Figure 3.**
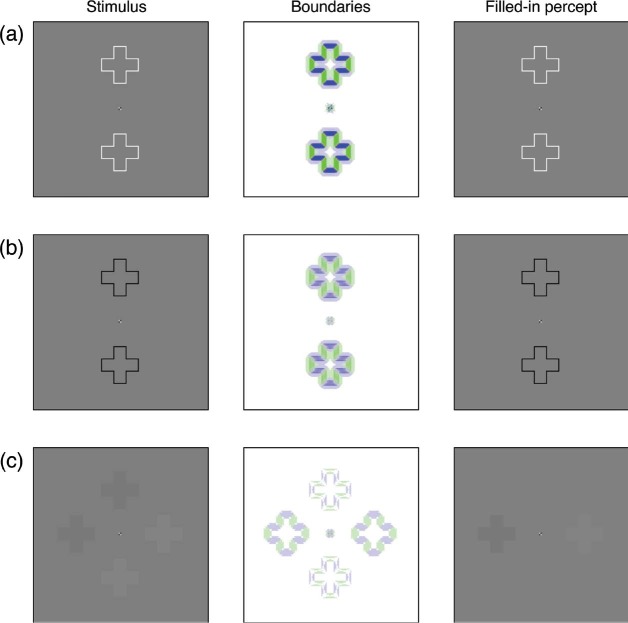
The model reproduces the observations of Anstis and Greenlee (2014) for their Movie 2. Each row corresponds to a fixed time in the simulation. Each column describes the stimulus (left), the response of oriented boundary signals (middle), and the filled-in percept (right). (a) Shows the model's behavior early in the adaptation phase of the flickering outline crosses. (b) Shows that contour adaptation weakens the strength of the boundaries, although they are still strong enough to support visibility of the flickering outline cross. (c) Shows that when the flickering outlines are replaced by cross shapes that are slightly darker or brighter than the gray background, the adapted boundaries are too weak to support filling-in. The faint boundaries at the top and bottom are of orientations opposite what would normally be induced by the surfaces. The test stimuli in (c) may be too faint to be legible on a printed copy of the manuscript; they are more visible in the electronic version of the manuscript and in Movie 1.

Figure 1 shows that the color opponent activities and the outputs from the gated dipole circuit feed into the Feature Contour System's filling-in stage. Here, the orientation signals act as boundaries to prohibit color signals from spreading to nearby locations. For example, an active vertical boundary at a pixel will prohibit color signals at that pixel from spreading horizontally. In most cases, the boundary signals correspond to edges in the image and thereby produce distinct filling-in domains that correspond to the elements in the original image. This process is demonstrated in Figure 3, which shows the input image, oriented boundary signals, and filling-in stage at three moments in time for a stimulus sequence that corresponds to Movie 2 in Anstis and Greenlee (2014). The contour adaptation stimulus consists of two outline cross shapes that alternately flicker between black and white. An early image during the adaptation sequence is a white outline cross on a gray background. Such an image produces strong responses from the oriented boundary stage of the model, as shown in the middle column of Figure 3a (a green pixel corresponds to a vertical boundary and a blue pixel corresponds to a horizontal boundary; the intensity of the color indicates the strength of the boundary signal). The effect of contour adaptation is visible by comparing the boundaries in Figure 3a with those in Figure 3b, which shows the final image during adaptation (a black outline cross). Although the boundaries are still fairly strong, they are much weaker than at the start of adaptation. At both time moments, the boundaries follow the general shape of the stimulus, so the boundary signals effectively trap the color signals of the outline shape and thereby the filling-in stage accurately represents the stimulus.

The next section shows that contour adaptation in the model has an effect on the filling-in of a subsequently presented stimulus that closely matches the observations of Anstis and Greenlee (2014).

## 3 Simulations of the Anstis and Greenlee (2014) observations

Figure 3c shows the model's behavior at the frame of the movie just after the adaptation sequence; here, the adapting outline contours are replaced with cross-shaped test stimuli having luminance values slightly above or below the background gray. For the crosses on the left and right sides of the image, the boundary contours follow the general shape of the stimuli. They are weak because the stimuli have low contrast with the background, but the boundaries sufficiently constrain the filling-in stage to accurately represent the stimuli as being distinct from the background. However, for the test crosses on the top and bottom parts of the image, the adaptation to the flickering outlines has weakened the boundary cells that would typically respond to such stimuli. Many of the weak boundary signals visible at the top and bottom locations of Figure 3c actually represent orthogonal orientations, relative to the edges of the test stimulus crosses. These orthogonal orientations are the after-responses indicated in Figure 2. Vertical boundaries (green pixels in Figure 3) prevent color/brightness information from flowing horizontally but they allow colour/brightness information to flow vertically. Likewise, horizontal boundaries (blue pixels in Figure 3) allow color/brightness information to flow horizontally. As a result, the colors of the interiors of the top and bottom crosses merge with the gray background. As the third column of Figure 3c shows, the net effect is that test crosses located at the positions subjected to contour adaptation become invisible. Movie 1 (movies may be found at http://i-perception.perceptionweb.com/journal/I/volume/6/article/i0684) demonstrates the model's behavior for the full simulation; it should be compared to Movie 2 in Anstis and Greenlee (2014).

Similar model properties explain most of the other stimulus conditions described by Anstis and Greenlee (2014). Movie 2 shows that the model correctly accounts for the observation that adaptation to a flickering surface with a blurred edge does not induce erasure of a subsequently presented test shape, even while adaptation to a flickering outline figure does produce erasure. The blurred edges of the adapting surface do not generate boundaries as strong as the outline figure and thereby produce less adaptation; as a result, the test stimulus generates boundaries that are strong enough to support a percept at the filling-in stage. The simulation results should be compared to Movie 3 in Anstis and Greenlee (2014).

Movie 3 shows that the model is sensitive to the relative size of the adapting outline stimulus and the size of the test stimulus. When the outline and test stimuli differ in size, unadapted cells respond to the edges of the test stimulus and thereby support it at the filling-in stage. When the adapting outline and test stimuli are of the same size, the neurons responsible for coding the boundaries of the test stimulus are weakened by the previous adaptation. The simulation results should be compared to Movie 4 in Anstis and Greenlee (2014).

The model does not do well for every stimulus sequence considered by Anstis and Greenlee (2014). Movie 4 shows the model's behavior when the adapting stimulus is a flickering half-square outline followed by a faint filled square. As Movie 5 in Anstis and Greenlee (2014) demonstrates, the perceptual experience of such a sequence is that the unadapted side of the surface appears nearly normal, while the adapted side fades into the gray background. In contrast, the model produces a much stronger erasure effect whereby all but a few brightness signals on the unadapted side disappear into the background. This poor match of the model likely indicates some misspecification of the filling-in process. In the model, a closed set of boundary contours is required to trap brightness signals. A gap on only one side is enough to cause a stimulus to blend into the background. Clearly, this aspect of the model is not correct, and similar problems have been identified in previous work (Francis & Ericson, 2004; Francis & Schoonveld, 2005; Van Horn & Francis, 2007; Kim & Francis, 2011), but a solution remains elusive.

Movie 5 shows that the model is successful in accounting for the properties of Movie 6 in Anstis and Greenlee (2014). Here, a flickering vertical line produces contour adaptation. When a subsequent bipartite surface appears, the vertical edge separating the two sides is too weak to separate the filling-in regions and the two sides appear to have the same brightness.

Movie 6 shows that the model also accounts for the percept of brightness pyramids described in Movie 7 of Anstis and Greenlee (2014). Here, the central square is much brighter/darker than the gray background, but is only modestly different from the surrounding pyramid step. An adapting stimulus defined by flickering contours at the step edges substantially reduces the perceived contrast of all the pyramid steps, even though the central step has a high contrast with the background. Anstis and Greenlee (2014) observed that producing full invisibility of the pyramids required 30 s of adaptation, but the model produces the effect more quickly. This difference may reflect the model simulation having perfect fixation (no simulated eye movements).

Movie 7 shows that the model has partial success for adapting contours on the perception of test annuli. Large or small flickering outline squares matched the inner or outer edges of bright or dark test annuli, as for Movie 8 in Anstis and Greenlee (2014). In agreement with their observations, the small adapting contours weaken the boundary separating the interior of an annulus from the surrounding region, which leads to a percept of a filled shape (without a perceived hole). When the adapting contour matched the outer edge of the annuli, Anstis and Greenlee reported that the outer surface of the annulus disappeared, and the model also produces this erasure. However, Anstis and Greenlee also noted that the hole of the (now erased) annulus appeared different than the gray background, presumably due to brightness contrast. The model simulation does not produce a visible hole. The model limitation is easily identified: the current simulation does not include mechanisms for brightness contrast. This deficit is specific to the current simulation (which was designed to account for scene fading, where brightness contrast effects did not play a role), rather than being a general problem for the model (e.g., see Grossberg & Todorovic, 1988 for a description of how the model accounts for brightness contrast effects). A modified simulation should reproduce the observations of Anstis and Greenlee (2014) for these stimuli.

Anstis and Greenlee (2014) also described two other conditions that explored how the effects of erasure for annuli influenced perceived pop-out of a set of stimuli. The current model simulation does not consider these conditions because the reported percepts seem to require brightness contrast effects.

Finally, Anstis and Greenlee (2014) reported that contour adaptation to rectangle outlines could lead to the erasure of gray horizontal bars on a vertically striped background. This author found that their Movie 11 did not produce the effect described in their text, but this discrepancy may reflect differences in the computer monitor or difficulties across observers for describing percepts of peripheral stimuli. The stimulus sequence was not simulated in the model, but the described effect seems generally consistent with the model's mechanisms.

## 4 Simulations of the Robinson and de Sa (2012, 2013) findings

Robinson and de Sa (2013) measured polarity discrimination of a luminance target after adaptation to induced flicker. Conceptually, the stimuli in Robinson and de Sa are similar to those in Anstis and Greenlee (2014), with the main difference being that the flicker stimulus was not an outline contour but a large annulus. Similar to the report of Anstis and Greenlee's Movie 4 (the present article's Movie 3), Robinson and de Sa found that contrast thresholds for the target stimulus were increased when the inner edge of the annulus abutted the location of the target stimulus, but that the contrast thresholds were low if the target was smaller than the inner edge of the flickering annulus.

**Figure 4.**
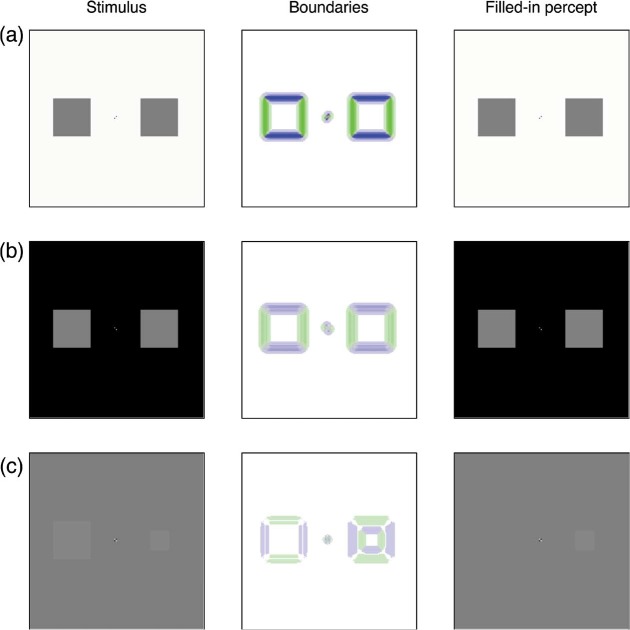
The model reproduces the effects reported by Robinson and de Sa (2013). Each row corresponds to a fixed time in the simulation. Each column describes the stimulus (left), the response of oriented boundary signals (middle), and the filled-in percept (right). (a) Shows the model's behavior early in the adaptation phase of the flickering two-holed annulus. (b) Shows that contour adaptation weakens the strength of the boundaries generated at the edge of the annulus holes, although they are still strong enough to support visibility of different regions of the image. (c) Shows that when the flickering annulus is replaced by faint squares on a gray background, the adapted boundaries are too weak to support filling-in for the larger test square. The faint boundaries around the larger test square are of an orientation opposite what would normally be induced by the square. In contrast, the small test square generates boundaries at unadapted locations, so it is represented at the filling-in stage. The test stimuli in (c) may be too faint to be legible on a printed copy of the manuscript. They are more visible in the electronic version of the manuscript and in Movie 8.

A model simulation with stimuli similar to those used by Robinson and de Sa produced the images in Figure 4. The flickering two-holed annulus produces strong boundaries along its interior edges (Figure 4a). These boundaries weaken over the 6-s flickering adaptation period, but they remain strong enough to support distinct surfaces in the filling-in stage (Figure 4b). After flicker adaptation, a target stimulus that equals the size of the annulus hole (left) and a target stimulus half as large (right) is presented on a gray background (Figure 4c). The previous contour adaptation weakens the boundaries too much to support a representation of the equal-sized target (the boundaries displayed in Figure 4c for the larger target are orthogonal to the edges of the inducing stimulus). However, the boundaries that constrain the smaller-sized target are unadapted and the filling-in stage can represent the small-sized target as a separate region. Movie 8 shows the full simulation of this condition.

**Figure 5.**
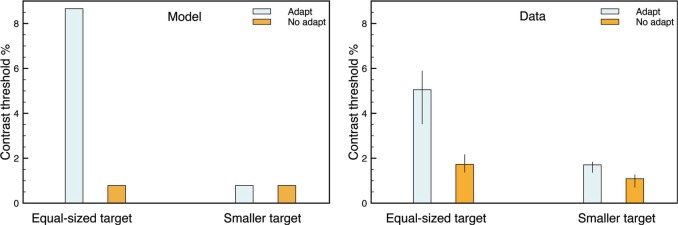
Contrast thresholds for the target stimulus after adaptation or not for an equal-sized or a smaller target. The pattern of thresholds for the model simulations (left) is similar to the experimental data (right) from Robinson and de Sa (2013).

Robinson and de Sa (2013) measured discrimination thresholds for their target stimuli (for judgments of whether the target was brighter or darker than the gray background). The simulation can be treated in a similar way, and Figure 5 compares the model's behavior to data from a representative observer in Robinson and de Sa (2013). For the model simulations, the stimulus sequence was repeated with varying intensities of the target to find the smallest value that produced a separate target representation at the model filling-in stage. The model's behavior is similar to the observer's data.

Robinson and de Sa (2013) argued that their findings were inconsistent with filling-in models of brightness perception that hypothesized a point-for-point representation of brightness information. The model of Francis and Kim (2012) is exactly the type of model they were addressing, but the simulations show that their experimental findings are entirely consistent with such a model. In fact, since the model was defined prior to their experiments, the findings can be taken as independent validation of the model properties. Robinson and de Sa (2013) ran additional conditions that are also consistent with the model's explanation. In their Experiment 1, they found very similar thresholds when they varied the size of the nonflickering annulus and the corresponding size of the target. Experiment 2 replicated Experiment 1 with a different measurement method. Experiment 3 found that adaptation had very little effect on a smaller test stimulus if the nonflicker region of the adapting stimulus varied during the adaptation period. These findings are entirely consistent with the model's explanation because an overlap of contours is required for the contour adaptation to have an effect on the filling-in stage. Such overlap only occurs when the adapting annulus interior and the target stimulus have nearly the same size.

**Figure 6.**
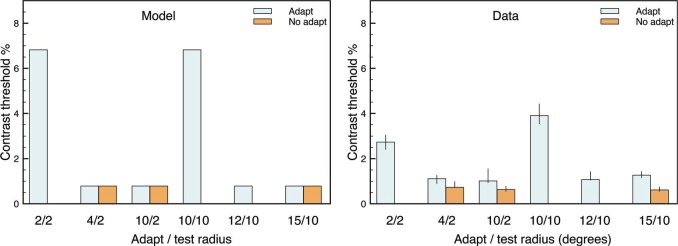
Contrast thresholds for the target stimulus after adaptation or not for different-sized adapting stimuli. The pattern of thresholds for the model simulations (left) is similar to the experimental data (right) from Experiment 1 of Robinson and de Sa (2012).

A similar argument accounts for the main findings of Robinson and de Sa (2012), which used different stimuli and different measures of test contrast thresholds. In their first experiment, the test stimulus was either a 2 or 10 degrees diameter flickering disk and the adaptor was a 2, 4, 10, 12, or 15 degrees flickering disk. As the data plot in Figure 6 demonstrates (for one representative observer), contrast thresholds were largest when the test and adaptor stimuli had the same size. The model plot in Figure 6 shows that the simulations reproduce this general pattern. Contrary to the model, the data do show a small difference between adaptation and nonadaptation conditions when the adaptation and test stimuli differ in size; and a revised model may need to include some direct form of flicker-related adaptation to account for this small effect. The model also produces larger contrast thresholds than the data for the same-sized stimuli, but this discrepancy may reflect differences in the calculation of the threshold. Robinson and de Sa (2012) measured the contrast threshold for 75% discrimination between a test disk that flickered four times from a test disk that flickered once, but the model simply identified the weakest contrast test stimulus (against the gray background) that produced a visible percept.

**Figure 7.**
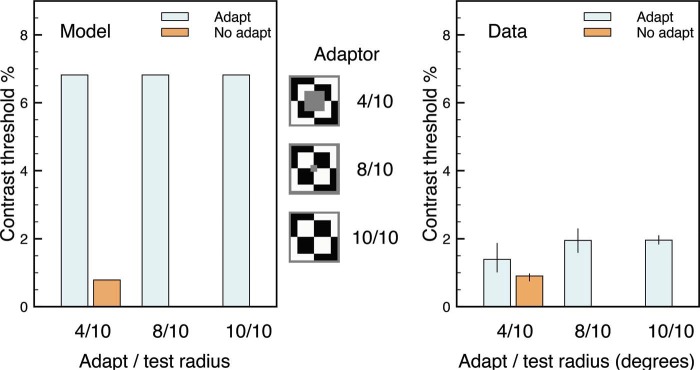
Contrast thresholds for the target stimulus after adaptation or not for different types of checkerboard adapting stimuli. For both the model and the data (Experiment 2 of Robinson and de Sa, 2012), all adapting stimuli show an increased threshold relative to a nonadapting stimulus.

Figure 7 shows that the model simulations also generally account for the findings in the second experiment of Robinson and de Sa (2012). Here, the test stimulus was fixed at 10 degrees and the adapting stimulus was a larger pattern that always had internal checkerboard contours at the outer edge of the test stimulus. What varied across adapting stimuli was the size (4, 8, or 10 degrees) of the innermost checks. The adaptor stimuli for the model simulations are schematized in Figure 7, and Movie 8 shows a full simulation for the 10/10 (adaptor/target sizes) condition. In qualitative agreement with the general findings of Robinson and de Sa (2012), the model produces a difference between adapt and no-adapt conditions and little difference among the different adaptation stimuli. The model suggests that the latter noneffect is because every adaptation stimulus includes a luminance contour at the same location as the edge of the test stimulus. Contour adaptation at this edge leads to an increased contrast threshold for the test stimulus. However, it is notable that the model predicts much bigger effects of contour adaptation than are found in the data. This discrepancy might indicate that a full account of the findings in Robinson and de Sa (2012) requires adaptation mechanisms for flicker rather than just for contours.

Although Robinson and de Sa (2012, 2013) designed their experiments to test adaptation effects of flicker, the model suggests that flicker plays only a minor role in most of their observed effects. Rather, the main effect of the flicker adaptation in their experiments is to weaken the boundaries generated by the flickering adaptation stimulus; that boundary weakening then affects the filling-in process that is needed to support a representation of the test stimulus as being distinct from the background.

## 5 Predicted absence of contour erasure when adapting to illusory contours

The model includes contour adaptation mechanisms because they generate orientation after-responses that function as “reset signals” to curtail excitatory feedback that would otherwise lead to unacceptably long visual persistence. Francis et al. (1994) used the model to explain the experimental finding that illusory contours have unusually long visual persistence (Meyer & Ming, 1988). In the model, contour adaptation predominately occurs before the development of illusory contours. The illusory contours do not cause adaptation and so the reset signals are only from the sparse inducing elements. Fewer reset signals allow the excitatory feedback loop to remain active for longer, which corresponds to longer persistence for illusory contours.

If the model's explanation of the persistence of illusory contours is valid, then it predicts that contour erasure should be absent, or much reduced, for an illusory adapting contour at the location of the test stimulus. The current simulation of the model does not include mechanisms for generating illusory contours, but since the prediction is that illusory contours will not produce adaptation, it is legitimate to consider the behavior of the model with stimuli that induce illusory contours.

**Figure 8.**
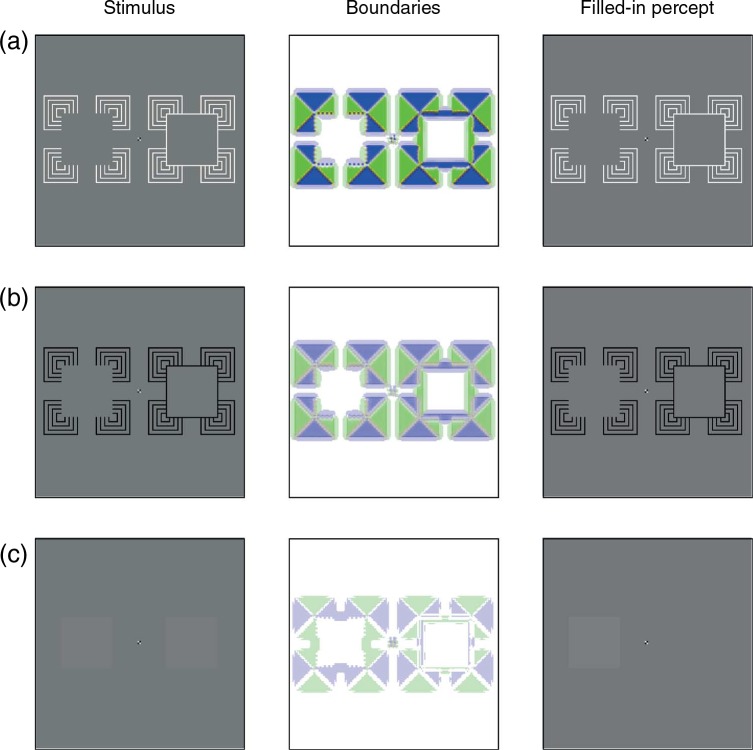
The model predicts that sparse illusory contour inducing stimuli will not produce contour erasure, even though they produce a vivid illusory contour at the location of the test stimulus. Each row corresponds to a fixed time in the simulation. Each column describes the stimulus (left), the response of oriented boundary signals (middle), and the filled-in percept (right). (a) Shows the model's behavior early in the adaptation phase. This version of the model does not produce illusory contours. (b) Shows that contour adaptation weakens the strength of the boundaries generated by the inducers. (c) Shows that the faint test square on the left (after adaptation to an illusory contour) can generate boundaries that support its representation at the filling-in stage, but the test square on the right (after adaptation to a real contour) cannot. The test stimuli in (c) may be too faint to be legible on a printed copy of the manuscript. They are more visible in the electronic version of the manuscript and in Movie 10.

Figure 8 shows the model's response to flickering cut-out concentric squares that produce an illusory square (left) and to identical inducers with a drawn contour (right). The illusory and drawn square contours are at the positions of subsequently presented test squares. The boundaries represent the orientations of the inducing elements, but they are very weak at the inducer line ends (even though they generate a much stronger illusory contour). The weak boundaries at the line ends do not produce much contour adaptation, so the test square at the location of the illusory contour produces sufficiently strong contours to constrain the filing-in process. In contrast, the real contour generates strong adaptation and leads to contour erasure of the test square on the right. Movie 10 allows the reader to verify that the illusory contour does not produce contour erasure.

## 6 Conclusions

With a few exceptions that are related to already known deficiencies, the model simulated by Francis and Kim (2012) to originally account for scene fading also accounts for the observations of Anstis and Greenlee (2014) on contour adaptation erasure and many of the findings from Robinson and de Sa (2012, 2013). Thus, these new reports validate the main properties of the model, especially the role of boundary contours in constraining a filling-in process. Finally, the model predicts that illusory contours do not produce adaptation that leads to contour erasure. This prediction seems to be validated by Movie 10.

An alternative theoretical approach hypothesizes that filling-in mechanisms are not needed to account for many brightness percepts. Instead, this alternative approach suggests that brightness perception derives from normalized outputs of oriented spatial filters that are summed across multiple scales (Blakeslee & McCourt, 2008). Simulations of such an approach do account for a wide variety of brightness illusions (Blakeslee & McCourt, 1999, Blakeslee, Pasieka, & McCourt, 2005), and it would be valuable to know if they can also account for the contour erasure effects that are so easily accommodated by a filling-in approach to brightness perception.

## Supplementary Material

Supplementary material

## Supplementary Material

Supplementary material

## Supplementary Material

Supplementary material

## Supplementary Material

Supplementary material

## Supplementary Material

Supplementary material

## Supplementary Material

Supplementary material

## Supplementary Material

Supplementary material

## Supplementary Material

Supplementary material

## Supplementary Material

Supplementary material

## Supplementary Material

Supplementary material
