# Supplementary figures and images for: Contour Erasure and Filling-in: Old Simulations Account for Most New Observations
Source: Iperception. 2015 Apr 1;6(2):116–26. doi: 10.1068/i0684 (PMC4950019; doi:10.1068/i0684)

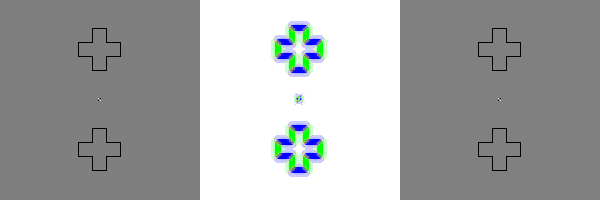

Supplement: Supplementary material [file Movie1.gif]

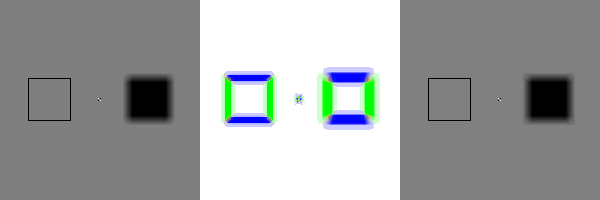

Supplement: Supplementary material [file Movie2.gif]

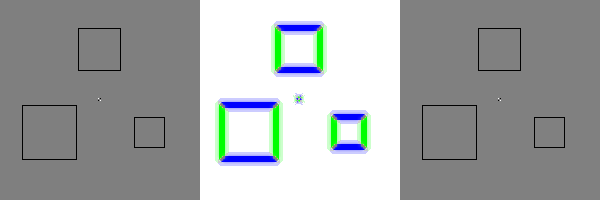

Supplement: Supplementary material [file Movie3.gif]

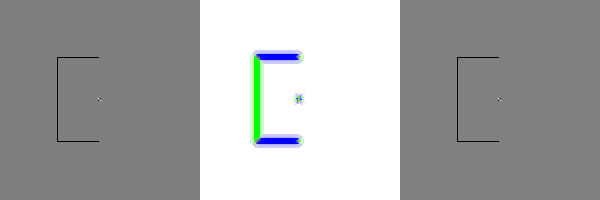

Supplement: Supplementary material [file Movie4.gif]

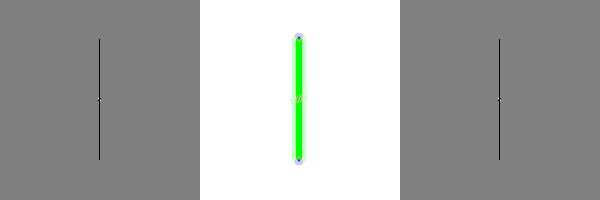

Supplement: Supplementary material [file Movie5.gif]

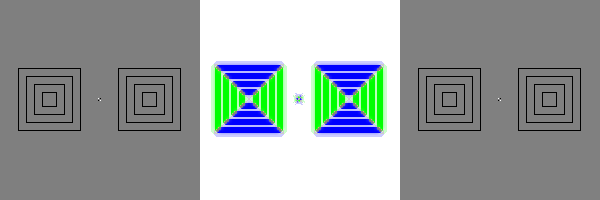

Supplement: Supplementary material [file Movie6.gif]

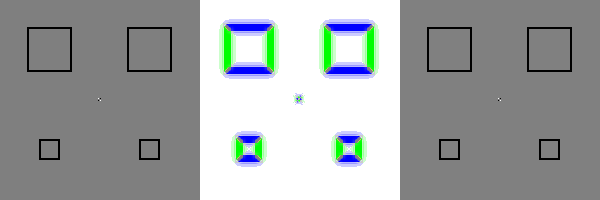

Supplement: Supplementary material [file Movie7.gif]

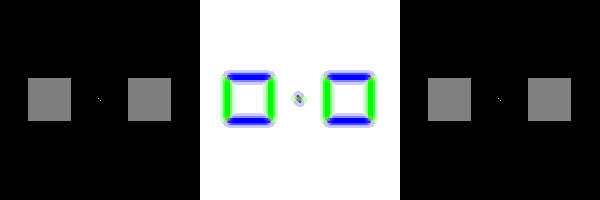

Supplement: Supplementary material [file Movie8.gif]

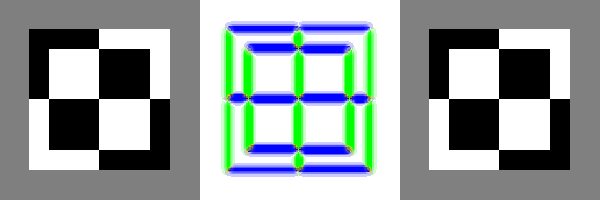

Supplement: Supplementary material [file Movie9.gif]

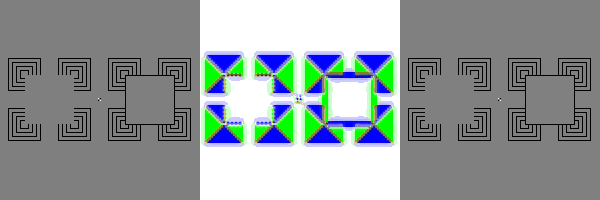

Supplement: Supplementary material [file Movie10.gif]
